# Oral Microbial Signatures of Tobacco Chewers and Oral Cancer Patients in India

**DOI:** 10.3390/pathogens12010078

**Published:** 2023-01-03

**Authors:** Shriya Sawant, Jinesh Dugad, Deepak Parikh, Sathiyaraj Srinivasan, Harinder Singh

**Affiliations:** 1 Department of Biological Sciences, Sunandan Divatia School of Science, NMIMS Deemed-to-be University, Vile Parle (West), Mumbai 400056, India; 2 Somaiya Ayurvihar-Asian Cancer Institute, Off Eastern Express Highway, Behind Everard Nagar, Somaiya Ayurvihar, Sion (East), Mumbai 400022, India; 3 Department of Bio & Environmental Technology, College of Natural Science, Seoul Women’s University, Seoul 139-774, Republic of Korea; 4 Gene Strand Technologies Pvt. Ltd., Chennai 600056, India

**Keywords:** oral cancer, dysbiosis, tobacco, biomarker, diagnosis, 16S rRNA

## Abstract

Dysbiosis of the oral microbiome has been found to play a key role in the genesis and progression of oral cancer (OC). Tobacco chewing, a risk factor for oral cancer, is also associated with oral dysbiosis. Since tobacco chewing is a lifestyle habit in the South Asian subcontinent, including India, and contributes to one-third of the global oral cancer burden; we aimed to identify the oral bacterial diversity of Indian oral cancer patients and tobacco chewers. We used 16S rRNA amplicon sequencing to study the composition of oral microbiota in OC patients and tobacco chewers in India and compared it with healthy controls. The abundance of predominant phyla, Firmicutes, and Bacteroidetes varied between the study groups. Our study identified Leptotrichia, Treponema, Lautropia, and Cardiobacterium as significantly enriched in tobacco chewers, whereas genera Pseudomonas, Capnocytophaga, and Mycoplasma were enriched in oral cancer, which could be potential biomarkers for the Indian population. Furthermore, the functional prediction revealed that genes involved in lipid biosynthesis and fatty acid elongation were upregulated in the oral cancer group, whereas those for the reductive TCA cycle were upregulated in the tobacco group. As the role of bacteria in oral cancer is becoming more evident, identification of bacterial diversity and biomarkers for tobacco chewers and OC patients can aid in the early diagnosis of OC in high-risk individuals.

## 1. Introduction

Cancer of the oral cavity is one of the most common malignancies, especially in Asia, where it contributes to approximately 66% of the global oral cancer (OC) burden, with an estimated 248,360 new cases and 131,610 deaths every year. The incidence of OC appears to be increasing worldwide, and this common cancer is most prevalent among males in India [1]. Despite advances in surgical methods, adjuvant radiation, and chemotherapy, the overall 5-year survival rate of OC patients is approximately 50–60%. OC treatment success rates can be improved by early identification and interdisciplinary therapy [2].

The most well-established risk factors associated with OC include chewing tobacco, betel quid, smoking cigarettes, alcohol consumption, and HPV-16/18 [3,4,5]. Over 90% of the worldwide smokeless tobacco usage burden is believed to be in Southeast Asia, with over 100 million individuals using smokeless tobacco in India and Pakistan alone [6]. Moreover, it was shown that the combination of smoking, drinking alcohol, and poor oral hygiene increases the risk of OC onset due to chronic inflammation and infection, which are the main factors in cancer pathogenesis, influencing the resident microbiota involved in the oral environment’s homeostasis [7,8]. Several metagenomic investigations of the microbiome have revealed microbial pattern changes in OC, which further vary depending on the stage of OC, malignant lesions, and diseases of the oral cavity, according to reports published [9].

Along with dysbiosis in OC, reports also suggest bacterial alterations due to tobacco chewing, thereby making an individual prone to bacterial infections by inducing bacterial virulence, deregulation of host immune functions, and physiological and structural changes in the human oral cavity [10]. Increased abundance of pathogenic bacterial genera such as Fusobacterium, Cardiobacterium, Synergistes, Selenomonas, Haemophilus, and Pseudomonas has been observed in tobacco users, depicting early acquisition and colonization of pathogens in oral biofilms due to tobacco exposure [11]. Even though most OC cases arise from the Indian subcontinent and tobacco chewing is a common lifestyle habit associated with OC in the population, there is a dearth of information on the microbiome in Indian groups of subjects. Especially, the microflora in the oral cavity of healthy individuals, tobacco chewers, and oral cancer patients has not been investigated.

In the current study, we aim to identify the bacterial diversity in the oral cavity of OC patients and long-term tobacco chewers from India. We hypothesize that the variations in oral microbiomes between tobacco chewers, OC patients, and healthy people are expressed in oral rinse samples, which may be detected by 16S rRNA gene amplicon sequencing. These variations might subsequently be linked to cancer development and exploited as a biomarker panel to predict tobacco chewers with a high risk of OC in the Indian population in a clinical setting with effective diagnostic accuracy.

## 2. Materials and Methods

### 2.1. Subject Recruitment

A total of 120 participants in the study were divided into three study groups, consisting of 40 participants each healthy controls (C), patients suffering from oral squamous cell carcinoma (OC), and long-term tobacco chewers (T). The sample size was calculated using power analysis. Individuals without any documented disorders in the oral cavity, as determined by earlier clinical evaluation, were considered healthy controls. Participants categorized as long-term tobacco chewers were those who had been chewing tobacco for at least 5 years. Biopsy and pathology results validated all diagnoses among OC participants. The clinical examination of the participants’ oral cavities was performed by a maxillofacial prosthodontist and a surgical oncologist. At the time of sample collection, the participants were devoid of any antibiotic treatment for a week prior to sample collection. Exclusion criteria included individuals under the age of 18, those medically compromised/unfit to give consent, subjects who were completely edentulous, and those who received oncotherapy earlier. All the samples were collected in the period from January 2018 to March 2020, in Mumbai, India. The oral cancer samples were collected from patients admitted at Somaiya Ayurvihar-Asian Cancer Institute, Mumbai.

The work described has been carried out in accordance with the Code of Ethics of the World Medical Association (Declaration of Helsinki). Medical history, age, gender, employment, cigarette and alcohol consumption habits, and general oral hygiene questions were all documented for participating individuals. All individuals provided written, informed permission prior to the sample collection. For the study, ethics approval was obtained from SVKM’s Institutional Ethics Committee (NMIMS/IEC/008/2016) and the Ethics Committee of K. J. Somaiya Medical College and Hospital, Mumbai.

### 2.2. Sample Collection, DNA Isolation and Sequencing

Oral rinse samples were collected from study participants as mentioned earlier [12,13]. Briefly, during the sample collection procedure, patients were asked to rinse their mouths for 30 s with sterile normal saline and spit into a sterile tube, of 50 mL. Participants were advised to abstain from eating, drinking, and doing oral hygiene procedures for at least one hour before sample collection. Salivary samples were collected in well-labeled sterile falcon tubes, stored at 4 °C, and processed within 48 h.

DNA extraction, V6–V8 hypervariable region amplification, sequencing, and processing of reads have been carried out as mentioned in our recent publication [13]. DNA was isolated using the Invitrogen PureLink™ Genomic DNA Kit (Cat no. K182002), according to the manufacturer’s recommendation. The PCR amplification of bacterial 16S rRNA hypervariable region V6-V8 was carried out using primers B969F (ACG CGH NRA ACC TTA CC) and BA1406R (ACG GGC RGT GWG TRC AA). The whole sequencing process was performed using Illumina (Illumina, San Diego, CA, USA), and MiSeq libraries were quantified and then subjected to 300-nucleotide paired-end multiplex sequencing on an Illumina MiSeq sequencer.

### 2.3. OTU Assignment and Diversity Analyses

The quality of the reads from the sequencer was assessed using FASTQC. The resulting pairs of reads from each sample were merged to obtain longer reads to improve the quality of reads (Phred score Q > 30) and taxonomy classification using VSEARCH. The standard QIIME2 (v. 2021.2) pipeline was used to analyze microbial diversity [14]. A closed reference-based OTU selecting technique, with 97% sequence similarity to the Greengenes database (gg_13_5), was utilized to cluster readings into operational taxonomic units (OTUs) and assign taxonomy to the OTUs at different taxonomic levels.

QIIME2 was used to assess alpha and beta diversity indices. Alpha diversity was assessed by indices such as ACE indicator, Chao1 index, Goods coverage, observed OTUs, pielou_e, Shannon index, and Simpson index. Whereas, beta diversity was assessed using phylogenetic (weighted and unweighted) and non-phylogenetic (Bray-curtis and Jaccard) Linear Discriminant Analysis (LDA) matrices and plots created using PhyloToAST [15].

### 2.4. Identification of Biomarkers and Prediction of Metagenomes

In order to identify the potential biomarker, LDA effect size (LEfSe) (https://huttenhower.sph.harvard.edu/galaxy/) (accessed on 1 May 2022) was performed to find out the differentially enriched taxa among the groups. The threshold for discriminative features was set to 2.0, and the results were displayed in a cladogram and histogram. The functional prediction of microbiota was performed with PICRUSt2 to obtain MetaCyc pathway abundances between the study groups.

### 2.5. Statistical Analyses

The relative abundance of bacteria and alpha diversity indices were compared and displayed using GraphPad Prism 8.0.2 (GraphPad Software, Inc., La Jolla, CA, USA). A one-way ANOVA followed by Tukey’s multiple comparison test was performed to evaluate the significance of alpha diversity indices. MANOVA/Wilks lambda was used to test for the significance of LDA clustering. The Lda Effect Size (LEfSe) was analyzed using the Kruskal Wallis test. The statistical analysis of predicted pathways obtained after PICRUSt2 in between the groups revealed significant findings using STAMP (version 2.1.3) after testing using Student’s t-test followed by Bonferroni correction. In all mentioned tests, a *p*-value < 0.05 was considered statistically significant.

## 3. Results

### 3.1. Characterization of Study Participants

The study cohort was composed of 40 participants belonging to each study group, i.e., healthy controls (C), long-term tobacco chewers (T), and histopathologically confirmed oral squamous cell carcinoma patients (OC). The clinical characteristics of the participants are included in Table S1 (Supplementary Materials). Of these 120 samples, 3 samples from the control, 1 sample from the tobacco group, and 4 samples from the OC group failed the sequencing procedure, and therefore their data is not included in the results below. The 16S rRNA amplicon sequencing data from this study have been deposited in the NCBI BioProject under accession number PRJNA751046.

### 3.2. OTU Assignment and Taxonomic Analyses of Bacterial Diversit

A total of 6,296,186 sequencing reads, ranging from 5458 to 155,742 per sample, were generated from the V6-V8 hypervariable region of the 16S rRNA gene. After strict quality and size filtering, 5,407,163 reads were retained, with an average of 48,278 reads per sample, and assigned to 6733 OTUs using the Greengenes database (gg_13_5). Rarefaction curves demonstrate that a species richness plateau (up to 500 OTUs) was reached in approximately 5000 readings per sample. To minimize sample variability, approximately 5000 reads were chosen as the minimum sampling depth to estimate diversity. Furthermore, the shape of the species accumulation curve derived from our dataset indicates that the community was well sampled because the specimens we gathered held significant information regarding total species richness.

**Figure 1:**
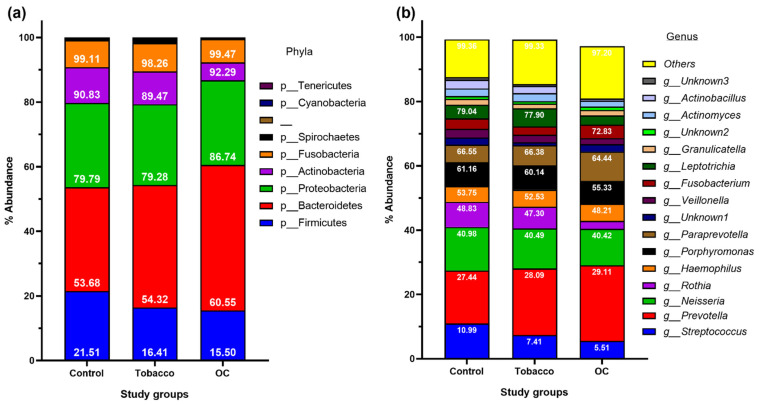
Oral bacterial profiles of healthy, tobacco chewing, and oral cancer patients in India at (**a**) Phyla-level and (**b**) Genus-level.

**Figure 2:**
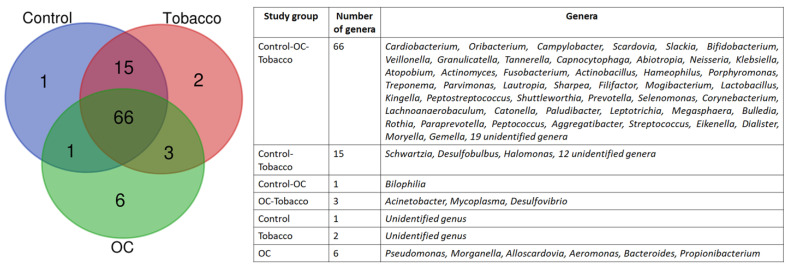
Venn diagram depicting the common and unique number of bacteria.

Overall, these OTUs were assigned to 9 phyla, 17 classes, 30 orders, 55 families, and 94 genera. Among the 5 most abundant phyla, Bacteroidetes dominated in all three groups, followed by Proteobacteria (Figure 1a). The next dominant phyla were Firmicutes, followed by Actinobacteria and Fusobacteria. The abundance of phyla consisting of Gram-negative organisms (Bacteroidetes and Proteobacteria) was higher in OC and tobacco samples than in healthy individuals, whereas that of Gram-positive organisms (Firmicutes and Actinobacteria). The five most abundant genera observed in all groups were Streptococcus, Prevotella, Neisseria, Rothia, and Haemophilus, which constituted up to 50% of total abundance at the genus level in all 3 study groups. The abundance of major genera Streptococcus, Neisseria, Rothia, Veillonella, and Leptotrichia was higher in the control population, followed by the tobacco group, and least in the OC group, whereas that of Prevotella, Haemophilus, Fusobacterium, Capnocytophaga, and Aggregatibacter was higher in OC and decreased in the control population (Figure 1b). When examined closely, the genera Pseudomonas, Morganella, Alloscardovia, Aeromonas, Bacteroides, and Propionibacterium were found only in the OC group (Figure 2).

### 3.3. Microbial Biomarkers in Control, Tobacco and OC Individuals

**Figure 3:**
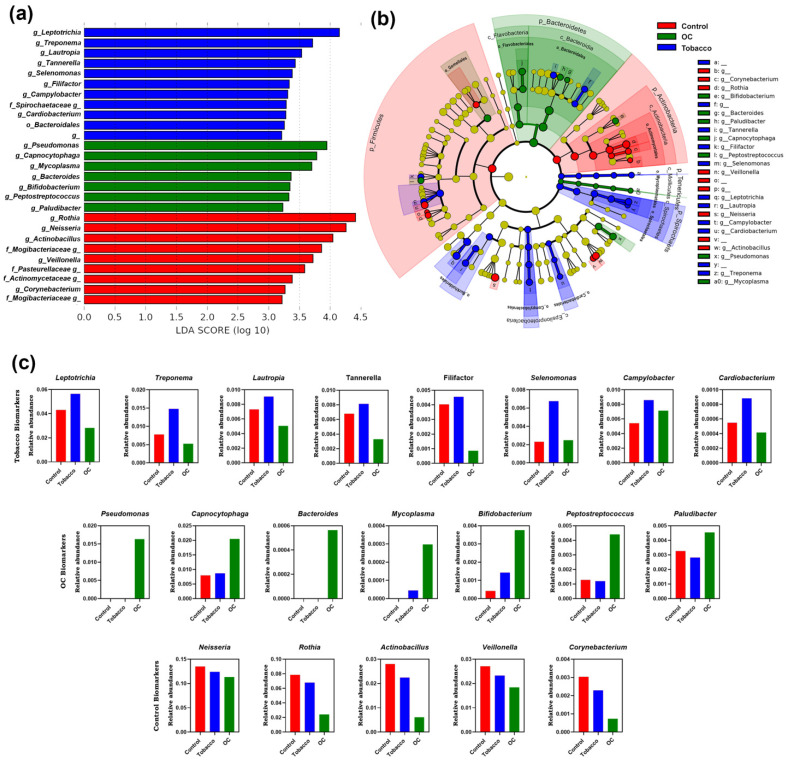
Distinct taxa were identified in the C, T, and OC groups using LEfSe analysis. (**a**) LDA scores showed significant bacterial differences within groups at the genus level; (**b**) a Cladogram was constructed using the LEfSe method to indicate the phylogenetic distribution of bacteria that were remarkably enriched in the control, tobacco, and OC groups; (**c**) and the mean relative abundance of biomarker taxon across all study groups.

The unique bacterial community composition associated with the oral rinse was investigated using LEfSe analysis to compare the relative abundance of taxa across the C, T, and OC groups (Figure 3a). A total of 27 bacterial genera were observed to be different in the 3 study groups. Leptotrichia, Treponema, Lautropia, Tannerella, Selenomonas, Filifactor, Campylobacter, and Cardiobacterium were identified as potential biomarkers for the tobacco group. On the other hand, Pseudomonas, Capnocytophaga, Mycoplasma, Bifidobacterium, Peptostreptococcus, and Paludibacter were associated as biomarkers for OC. Bacteria belonging to the genera Rothia, Neisseria, Actinobacillus, Veillonella, and Corynebacterium were identified as potential biomarkers for the control population. Furthermore, the cladogram could be used to determine the branch evolution connection, which also depicts the biomarkers identified in the OC group mainly belonging to phyla Bacteroidetes (Figure 3b).

### 3.4. Diversity of Microbiota Associated with Tobacco Chewing and OC

**Figure 4:**
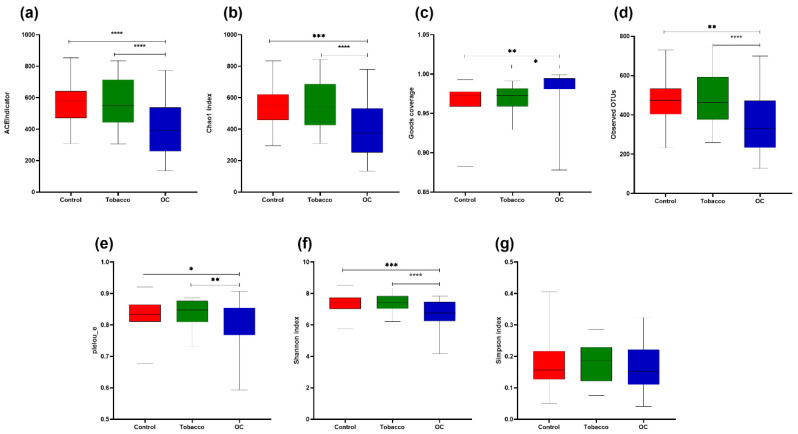
Alpha diversity indices for the study groups; (**a**) ACE indicator; (**b**) Chao1 index; (**c**) Goods coverage; (**d**) Observed OUT’s; (**e**) pielou_e; (**f**) Shannon index; (**g**) Simpson index. (* *p* < 0.05, ** *p* < 0.01, *** *p* < 0.001. **** *p* < 0.0001).

**Figure 5:**
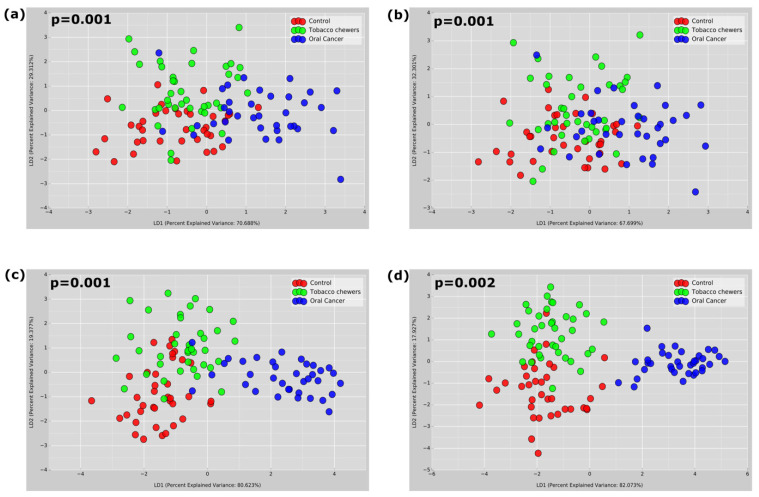
Beta diversity LDA plots depicting sample diversity between groups; (**a**) Bray- Curtis plot; (**b**) Jaccard matrix; (**c**) Unweighted Unifrac matrix; (**d**) Weighted Unifrac matrix.

Alpha diversity matrices were generated using observed OTUs, the Ace index, Chao1, Goods coverage, Shannon and Simpson indices, and Pielou_e to understand the species richness and diversity of the samples (Figure 4). Good’s coverage was >96% for sequences in all the study groups, indicating that the sequences measured in each sample represented almost all the bacterial sequences in the sample. A significantly higher number of mean OTUs was observed in tobacco chewers and control populations compared to the OC group. Other alpha diversity indices, such as hose of species richness (ACE/Chao1) and diversity index (Shannon index) also depict statistically higher alpha diversity observed in tobacco chewers and control populations as compared to the OC group, thereby indicating the lowest alpha diversity in the OC group. Beta diversity was studied using various parameters depicted in Figure 5. To advocate for the beta-diversity results obtained to assess community dissimilarity, the Bray-Curtis matrix, the Jaccard matrix, and the Weighted and Unweighted Unifrac matrices were compared (Figure 5). All beta-diversity matrices affirm the bacterial communities in the OC group and the controls-tobacco group clustered discretely, suggesting the overall structures of the bacterial communities in the groups were significantly different.

### 3.5. Functional Prediction of Bacterial Communities Related Tobacco Chewing and OC

**Figure 6:**
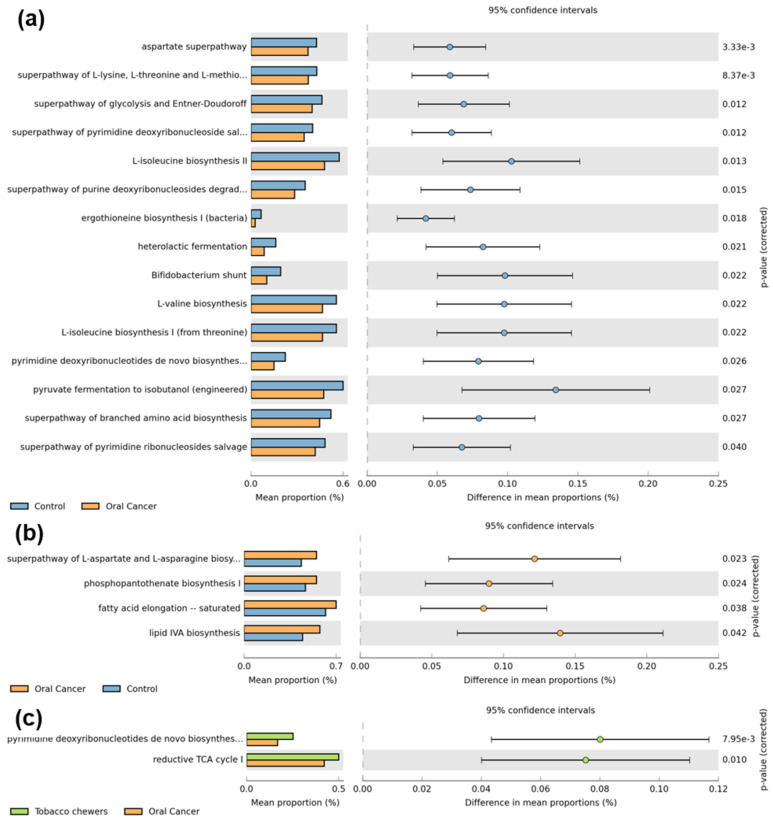
Prediction of microbial gene functions among distinct mutational signature clusters of OC. (**a**) depicts significant pathways upregulated in healthy control samples as compared to oral cancer samples, which includes amino acid biosynthesis (aspartate, lysine, methionine, threonine, isoleucine and valine), sugar fermentation (glycolysis, Entner Doudoroff, pyruvate) and pyrimidine salvage and biosynthesis pathways. (**b**) depicts pathways significantly upregulated in oral cancer samples as compared to healthy control samples, which include co-enzyme A, aspartate, asparagine and lipid biosynthesis pathways. (**c**) depicts pathways upregulated in the tobacco group as compared to the oral cancer group which include reductive TCA and pyrimidine biosynthesis pathways.

We used the Phylogenetic Investigation of Communities by Reconstruction of Unobserved States (PICRUSt2) method to envisage oral microbial roles linked to the formation of OSCC, and MetaCyc pathways were constructed for the study groups. PICRUSt2 estimates which gene families are present using an extended ancestral-state reconstruction technique, and then joins gene families to provide a comprehensive metagenome of the data. Significantly upregulated pathways related to amino acid biosynthesis (aspartate, lysine, methionine, threonine, isoleucine, valine), sugar fermentation (glycolysis, Entner-Doudoroff, pyruvate), and pyrimidine salvage and biosynthesis were detected in healthy controls as compared to the OC group (Figure 6). Conversely, pathways related to Co-enzyme A (*p* = 0.024), aspartate, asparagine (*p* = 0.023), lipid biosynthesis (*p* = 0.042), and fatty acid elongation (*p* = 0.038) were upregulated in the OC group as compared to controls. The tobacco group revealed upregulated pathways related to the reductive TCA cycle (*p* = 0.010) and pyrimidine biosynthesis (*p* = 7.95 × 10^−3^) as compared to the OC group.

## 4. Discussion

Numerous oral microbiome-based research studies have been conducted throughout the world to better understand bacterial dynamics in the context of diverse external factors and diseases, mainly cancer. However, the population of the Indian sub-continent is highly diverse in terms of ethnicity, culture, lifestyle, geographic location, and food. The Indian population is exposed to a wide variety of lifestyle factors, including tobacco chewing, smoking, and alcohol consumption, and ranks first in the incidence of males suffering from OC across the globe [16]. Thereby, the Indian population acts as a suitable demography to study the OC microbiome due to the high incidence rate as well as exposure to the risk factors. Handful studies regarding the Indian oral microbiome have been published, but this is the first report comparing the oral microbiome of healthy controls and tobacco chewers with OC patients.

In this study, we have analyzed a larger population group for accurate information regarding the study population compared to an earlier published report [13]. The present study reveals five phyla and 23 genera contributing to approximately 90% of the total oral microbiome composition. The abundance of Bacteroidetes and Proteobacteria observed was highest in the OC group as compared to the other two study groups. These phyla are composed mainly of Gram-negative bacteria, whereas Proteobacteria includes mainly Gram negative pathogenic bacteria [17]. The increased presence of Gram-negative bacteria in the oral microbiota of OC patients has been previously reported [18]. Apart from the five major phyla, the abundance of phyla Spirochaetes was highest in the tobacco group, and the presence of phyla Tenericutes was observed in tobacco and OC groups only, which could be attributed to the presence of periodontal pathogens in tobacco chewers and diseased conditions in OC [19]. The abundance of genera Streptococcus, Rothia, Veillonella, and Neisseria was found to decrease in individuals suffering from OC compared to those in healthy controls, owing to the mentioned genera being part of the healthy oral microbiota in humans. Therefore, their high abundance in controls is due to a healthy oral cavity, whereas their decreased abundance in OC and tobacco chewers could be due to dysbiosis in the oral cavity of the said individuals. On the other hand, genera such as Prevotella, Haemophilus, and Fusobacterium are known pathogens of the oral cavity, thereby justifying their increased counts in the OC and tobacco chewing [20]. The increased abundance of Prevotella and Fusobacterium in tobacco chewers leads to the synergistic activity of toxins from the bacteria and nicotine, thereby leading to detrimental health effects [10]. Fusobacterium spp. Has been linked to cell adhesion, tumorigenesis, epithelial-to-mesenchymal transition, inflammasomes, the cell cycle, and other aspects of oral cancer [21,22].

Along with being residents of the human body, some microorganisms can also cause host damage. Any kind of damage can cause inflammation, which is a defense mechanism to eliminate harmful metabolites and damaged tissues and is followed by the initiation of wound healing [23]. The use of smokeless tobacco is another source of tissue damage that can disrupt the wound-healing process. Recent studies have provided a hypothesis that human immunity has emerged as an entity that can control the damage exerted to host tissues by the inflammation process and also manage the microbes present inside and around the human body for nutrition [24]. Tobacco chewing, along with microbial dysbiosis, can lead to chronic inflammation that can initiate and progress toward the development of oral cancer. Because the role of microbiota and lifestyle habits such as tobacco chewing are linked to inflammation, identifying microbial biomarkers can help in the recognition of inflammation markers and related molecular pathways. Genus Leptotrichia, a biomarker for tobacco chewers, has been previously linked to tobacco chewing habits [25]. Leptotrichia and Campylobacter have been linked to the core OC microbiota [26]. Similarly, genera Treponema and Tannerella are well-known periodontal pathogens that play a crucial role in the formation of a red complex periodontitis [27]. Therefore, the increased abundance of these bacteria in the tobacco-chewing population can be attributed to a higher risk of periodontitis development in these individuals. The presence of other tobacco biomarkers, such as Lautropia, Filifactor, and Selenomonas, has been linked to the occurrence of OC in different populations [9]. Filifactor bacteria have been shown to secrete proinflammatory cytokines, activate specific oncogenes, and maintain an inflammatory state [28]. Although the genus Cardiobacterium has been associated with endocarditis and oral mucositis [29], we report a significant increase in the genus Cardiobacterium in tobacco chewers for the first time.

On assessing the OC microbiome, Pseudomonas and Bacteroides were found solely in the OC patients, previously reported as a part of OC microbiota [30] can be used for early clinical diagnosis by using simple, specific, non-invasive methods for identification purposes [12]. Pseudomonas can convert salivary nitrite to nitric oxide (NO), which modulates various cancer-related appearances such as apoptosis, cell cycle, angiogenesis, invasion, and metastasis [31]. Similarly, concurring with the previous study, Capnocytophaga and Peptostreptococcus were enriched in OC patients, whereas the abundance of Bifidobacterium is upregulated in our study in the OC group as opposed to previous reports [32]. Similar to increased abundances of tobacco and OC biomarkers, decreased populations of healthy control biomarkers can also be used to diagnose dysbiosis, thereby predisposing individuals to diseased conditions.

Apart from the biomarkers identified, a few genera, such as Acinetobacter, Mycoplasma, and Desulfovibrio, have been found only in the tobacco and OC populations. The proportions of Mycoplasma and Desulfovibrio were observed to be higher in oral cancer patients as compared to tobacco chewers, respectively, and are well reported [33]. Since Mycoplasma is already identified as a biomarker, and Desulfovibrio also shows similar patterns of existence, these can be an important choice of bacteria to monitor the initiation and progression of oral cancer in tobacco chewers. Similarly, bacteria belonging to the genus Morganella, Alloscardovia, Aeromonas, and Propionibacterium, along with Pseudomonas and Bacteroides have been identified only in the OC population. For the first time in our study, Aeromonas, Alloscardovia, and Morganella have been identified as part of the OC oral microbiota and therefore need to be studied in more detail. Furthermore, the predicted functions enriched in the OC samples depict increased lipid and fatty acid synthesis. These molecules have inflammatory functions and have been reported to initiate and aggravate oral cancer [34].

When the bacterial makeup of the three research groups is compared, it is observed that the abundance of major genera in tobacco chewers lies in between that of the control and OC populations. Similarly, beta diversity plots display the clustering of control and tobacco samples together, compared to OC samples that cluster away. Considering all of the parameters, it can be concluded that the composition of tobacco chewers is comparable to both the control and OC populations in several aspects, indicating the transitional phase of the tobacco chewers’ oral microbiota. Apart from OC, there are a few reports on the oral microbiota of oral potentially malignant disorders (OPMD). OPMD progresses to oral cancers through a series of histopathological stages, beginning with hyperkeratosis/hyperplasia and progressing to various degrees of dysplasia. Similar to the microbiota of tobacco chewers and the oral cancer identified in this study, the abundance of the phyla Bacteroidetes and Proteobacteria were higher, whereas that of Firmicutes was lower in the OPMD group. At the genus level, Alloprevotella, Leptotrichia, Fusobacterium, Campylobacter, Neisseria, Gemella, and Granulicatella were found in higher abundance in OPMD, similar to tobacco chewers and OC patients as compared to the control group reported in this study [26,35].

Based on the subject demographic, the study may have certain limitations. Male participants are more numerous in oral cancer and tobacco group than female participants. This is partly because males are more likely than females to use tobacco products, and more men than women are diagnosed with mouth cancer. Additionally, because age is a confounding risk factor for malignancies, including oral cancer, the study population includes participants in the OC group who are older than those in the control and tobacco chewing groups, potentially creating an age-related bias. The study may be limited by the inability to control the aforementioned variables; thus, this should be taken into account.

In conclusion, a compositionally distinct microbiota is identified using oral saline rinse in healthy, tobacco-chewing, and OC patients in the Indian population. Oral cancer is frequently thought to be a complicated illness caused by a number of interdependent host–environment interactions. As a result, using a single biomarker to identify oral cancer is exceedingly improbable. The present study used a non-invasive method for sample collection and NGS analysis for the identification of an array of oral microbial biomarkers, which can be useful for the early diagnosis of OC, especially in individuals susceptible to OC due to lifestyle habits such as tobacco chewing. Since the present study focused on the Indian population, where such information is scarce, this can serve as a reference and basis for future microbiome analysis and oral microbial biomarker studies related to oral cancer. This study provides the first epidemiological evidence for the association of Cardiobacterium in tobacco chewers and Aeromonas, Alloscardovia, and Morganella with OC. In addition to the presented data, it is necessary to investigate the role of differentially abundant taxa and discovered pathways in the development and progression of OC.

## Data Availability

The 16S rRNA amplicon sequencing data from this study have been deposited in the NCBI BioProject under accession number PRJNA751046.

## Supplementary Materials

The following supporting information can be downloaded at: https://www.mdpi.com/article/10.3390/pathogens12010078/s1, Table S1: Clinical characteristics of participants.
