# Supplementary material for: Oral Microbial Signatures of Tobacco Chewers and Oral Cancer Patients in India
Source: Pathogens. 2023 Jan 3;12(1):78. doi: 10.3390/pathogens12010078 (PMC9864012; doi:10.3390/pathogens12010078)
Supplement: Supplementary file 1 [file pathogens-12-00078-s001.zip › pathogens-2130058-supplementary.pdf]

Table S1: Clinical characteristics of participants

**Gender distribution**		**Control**	**Tobacco chewers**	**OC patients**
	Male	27	35	35
	Female	13	5	5
**Age distribution**		41 ± 10	43 ± 10	54 ± 11
**Lifestyle habits**	Tobacco chewing only	NA	30	16
	Tobacco + Smoking		2	2
	Tobacco + Alcohol		6	1
	Tobacco + Smoking + Alcohol		2	0
**Primary site of cancer**	Buccal mucosa	NA		17
	Tongue			9
	Mandible			7
	Retromolar trigone			1
	Lip			1
	Floor of mouth			2
	Maxilla			1
	Gingiva			1
	Alveolus			1
**Staging**	Stage II	NA		30
	Stage III			10

NA: Not Applicable to Control and Tobacco chewers
